# Ten-Year Trends in Sales of Alzheimer Disease Drugs in France Compared With Sales in Germany, Spain, and the UK

**DOI:** 10.1001/jamahealthforum.2022.2253

**Published:** 2022-08-05

**Authors:** Céline Ben Hassen, Rameen Tahir, Archana Singh-Manoux, Dejan Milic, Claire Paquet, Séverine Sabia, Julien Dumurgier

**Affiliations:** 1 Université Paris Cité, Inserm U1153, Epidemiology of Ageing and Neurodegenerative Diseases, Paris, France; 2 IQVIA, Paris, France; 3 Université Paris Cité, Cognitive Neurology Center, Lariboisiere–Fernand Widal Hospital, Assistance Publique–Hôpitaux de Paris, Paris, France

## Abstract

This cross-sectional study analyzes 10-year trends in sales of Alzheimer disease drugs in France compared with trends in the UK, Spain, and Germany.

## Introduction

Approximately 50 million people worldwide currently live with dementia, mainly Alzheimer disease (AD). Discovery of effective disease-modifying drugs remains elusive, and current AD pharmacotherapy relies on symptomatic drugs: acetylcholinesterase inhibitors^1^ (donepezil, rivastigmine, and galantamine) and antagonist of N-methyl-D-aspartate receptors (memantine).^2^ Use of these drugs is debated owing to limited efficacy. Health authorities in France raised concerns about their use in 2011 and 2016, leading to their removal from the list of reimbursed drugs in 2018.^3^ This cross-sectional study aimed to analyze 10-year trends in sales of AD drugs in France compared with trends in the UK, Spain, and Germany.

## Methods

Ethical approval was not required for this study because only market data were used. We followed the Strengthening the Reporting of Observational Studies in Epidemiology (STROBE) reporting guideline. The IQVIA MIDAS drug database^4^ was used to extract nationally representative sales of AD drugs between October 2009 and December 2019 in France, Germany, Spain, and the UK. Data from France were more granulated and included monthly vs quarterly sales with additional information on age of patients. The age distribution of the population in each country was extracted from national statistics sources. For France, estimates were expressed as monthly sale in age-standardized units (standard doses) per 1000 individuals in 3 age groups (60-74 years old, 75-84 years old, and ≥85 years old). For cross-country comparison, estimates were expressed as quarterly sale of units per 1000 individuals 60 years and older. We examined trends over time in sale of AD drugs using piecewise regression (R “segmented” package [R Foundation]). For further details, see eMethods in the Supplement.

## Results

**Figure 1.**
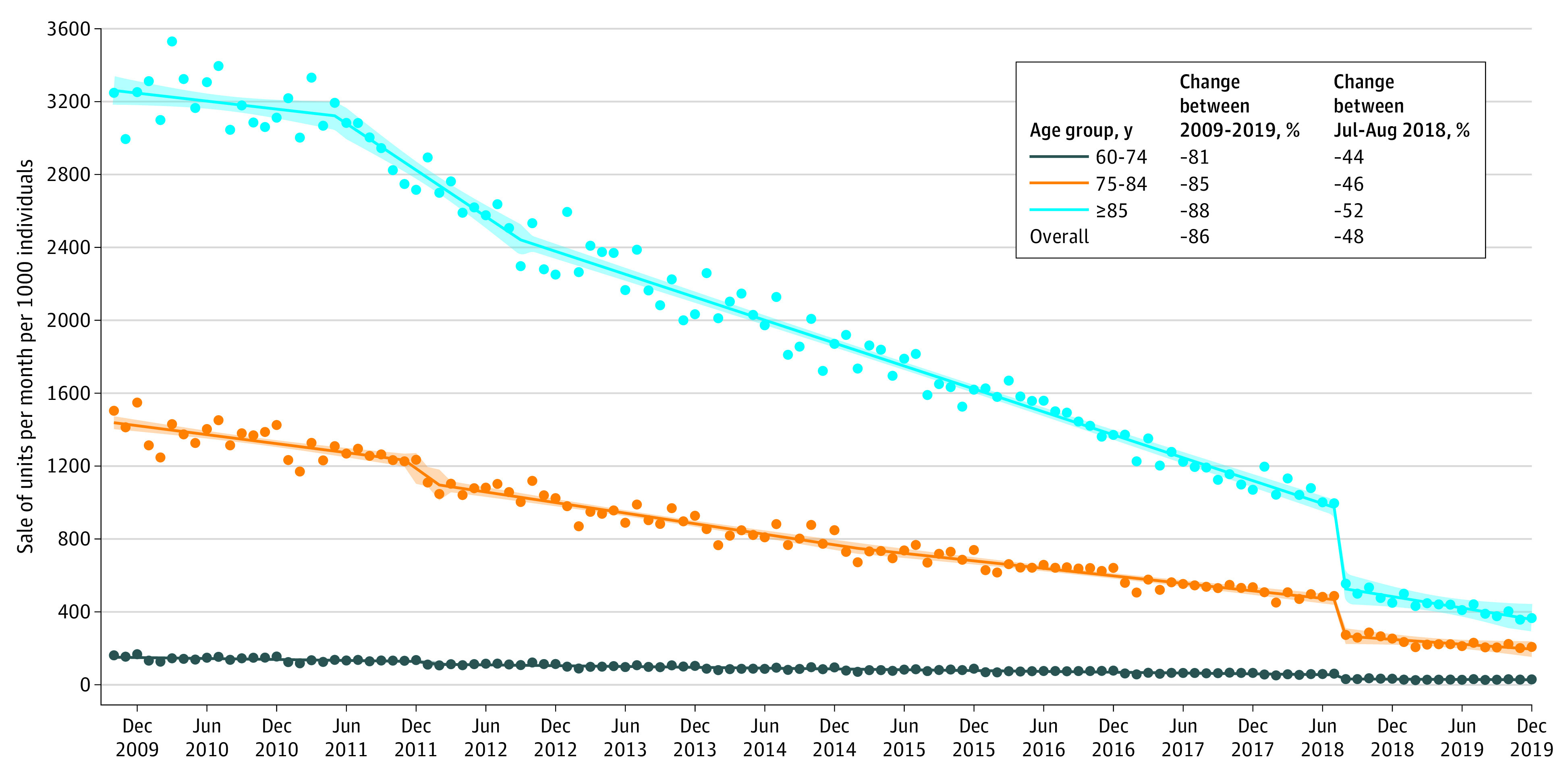
Sale of Drugs for Alzheimer Disease Between 2009 and 2019 as a Function of Age in France Data show standardized units sold per month per 1000 individuals within each age group. The period between July and August 2018 corresponds to the period of delisting Alzheimer disease drugs in France. Dots represent observed data, solid lines represent regression estimates, and shaded areas represent 95% CIs.

Figure 1 displays sale of AD drugs in France between October 2009 and December 2019 by age group, representing a total of 851 933 020 units sold during this period. The sales showed an 86% decrease overall over this period, ranging from 81% for those 60 to 74 years old to 88% for those 85 years and older. A marked decrease was observed first between 2011 and 2012, followed by a steady decrease up to the end of 2018. Between July and August 2018, the period corresponding to delisting of AD drugs in France, the sales dropped by 48%, which accounted for 18% of the total decrease.

**Figure 2.**
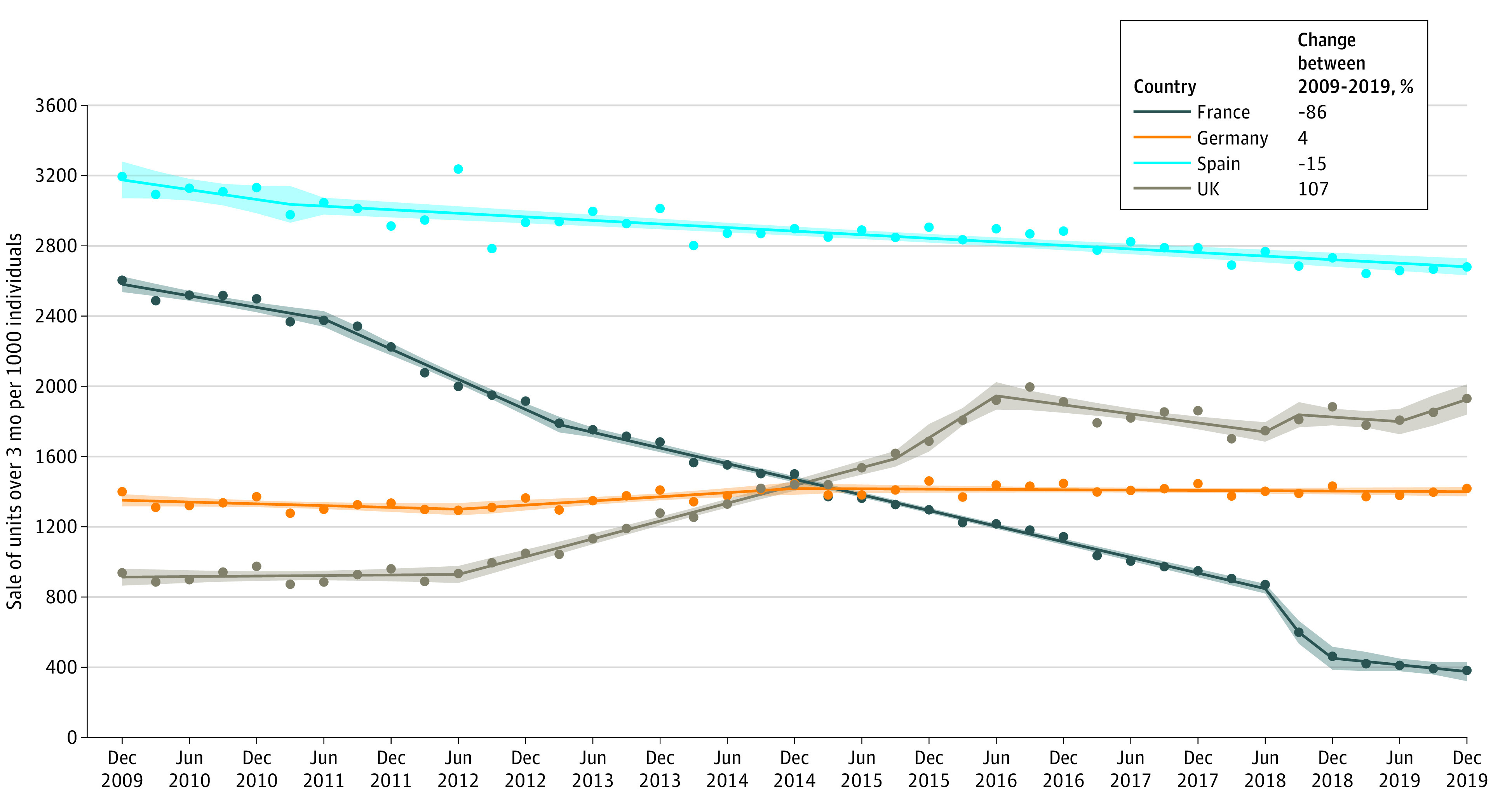
Sale of Drugs for Alzheimer Disease Between 2009 and 2019 in France, Germany, Spain, and the UK Data show aggregated units sold over 3 months per 1000 individuals, standardized on the population aged 60 years and older for each country. Dots represent observed data, solid lines represent regression estimates, and shaded areas represent 95% CIs.

Figure 2 shows the trends in quarterly sales of AD drugs between 2009 and 2019 in France, Germany, Spain, and the UK, representing a total of 1 002 051 040, 1 475 216 947, 1 520 259 094, and 1 052 354 772 AD drugs units, respectively. There were considerable differences in trend by country. While sales decreased by 86% over the 10-year period in France, they decreased by 15% in Spain, remained stable in Germany (4% increase), and increased by 107% in the UK.

## Discussion

In this cross-sectional study, analysis of nationally representative pharmacy retail data shows that the sales of AD drugs fell by 86% in France over 10 years, reflecting a considerable association with changes in health policy and prescription of AD drugs. Two previous studies based on nonnationwide representative data and focusing on the period following the delisting of AD drugs in summer 2018 also reported a drop in sales in France.^5,6^ The present study adds to the current evidence by showing that there has been a steady decline in sales of AD drugs, reflecting reduced prescribing, over the past decade, with the drop after the policy change in 2018 accounting for only 18% of the 10-year decline. The 10-year trend in France contrasted with trends observed in Spain (slight decrease), Germany (stable), and the UK (increase).

The limitations of this study include data on age of patients being available only in France and lack of data on individual trajectories of AD treatment. The reasons behind the difference in trends between countries and the effect on the natural course of disease require future research.

## Notes

Supplement. eMethods eReferences

## References

[1] Birks JS, Harvey RJ. Donepezil for dementia due to Alzheimer’s disease. Cochrane Database Syst Rev. 2018;6(6):CD001190. doi:10.1002/14651858.CD001190.pub3 29923184 PMC6513124

[2] Kishi T, Matsunaga S, Oya K, Nomura I, Ikuta T, Iwata N. Memantine for Alzheimer’s disease: an updated systematic review and meta-analysis. J Alzheimers Dis. 2017;60(2):401-425. doi:10.3233/JAD-170424 28922160

[3] Brief summary of the transparency committee opinion. Haute Autorité de Santé. October 2016. Accessed July 4, 2022. https://www.has-sante.fr/upload/docs/application/pdf/2017-05/dir24/aricept_exelon_reminyl_ebixa_summary.pdf

[4] Bortone B, Jackson C, Hsia Y, Bielicki J, Magrini N, Sharland M. High global consumption of potentially inappropriate fixed dose combination antibiotics: analysis of data from 75 countries. PLoS One. 2021;16(1):e0241899. doi:10.1371/journal.pone.0241899 33471786 PMC7817037

[5] Herr M, Ankri J, Diard C, Hiance-Delahaye A. Removal of drugs for Alzheimer’s disease from the list of reimbursable drugs in France: analysis of change in drug use, disease management and cognition using the National Alzheimer Data Bank (BNA). Drugs Aging. 2021;38(1):63-74. doi:10.1007/s40266-020-00817-3 33410119

[6] Noël V, Mouchoux C, Krolak-Salmon P, Novais T. Evolution in the dispensation of drugs for Alzheimer’s disease after removal from the list of reimbursable drugs in France. J Am Geriatr Soc. 2021;69(8):2350-2352. doi:10.1111/jgs.17151 33818771

