# Supplementary material for: Ten-Year Trends in Sales of Alzheimer Disease Drugs in France Compared With Sales in Germany, Spain, and the UK
Source: JAMA Health Forum. 2022 Aug 5;3(8):e222253. doi: 10.1001/jamahealthforum.2022.2253 (PMC9356313; doi:10.1001/jamahealthforum.2022.2253)
Supplement: Supplement. — eMethods eReferences [file jamahealthforum-e222253-s001.pdf]

Supplemental Online Content

Ben Hassen C, Tahir R, Singh-Manoux A, et al. Ten-year trends in sales of Alzheimer disease drugs in France compared with sales in Germany, Spain, and the UK. *JAMA Health Forum*. 2022;3(8):e222253. doi:10.1001/jamahealthforum.2022.2253

eMethods

eReferences

This supplemental material has been provided by the authors to give readers additional information about their work.

© 2022 Ben Hassen C et al. *JAMA Health Forum.*

eMethods

Data on AD drug sales contained were available up to the end of 2020 but we chose the observation period to be from 2009 to December 2019, excluding data from 2020 to avoid bias that could have arisen from differences in country-specific lockdown policies during the Covid pandemic.

Information relative to the age distribution of the population in each country was extracted from national statistics sources (Institut national de la statistique et des études économiques, Statistisches Bundesamt, Instituto Nacional de Estadística, and Office of National Statistics, respectively).[1,2,3,4]

In the dataset for France, we calculated the total units (standard doses) sold per month for all AD drugs combined (Donepezil, Rivastigmine, Galantamine, Memantine) as a function of age (every 1-year increment). Persons with missing data on age (1.8% of total sample) were excluded from the analysis. To account for changes in the age-structure of the population over the observation period we used direct standardization, with the 2009 population as the reference population. The analyses were undertaken for three age groups (60-74, 75-84, and ≥85 years). Final estimates represent the age-standardized sales of units of AD drugs per month per 1 000 individuals in each age group.

For cross-country comparisons, sum of units sold each quarter was divided by the number of adults aged 60 years and older in each country respectively for every year between 2009 and 2019 to take into account differences in the age-structure between countries and over time. Final estimates represent the sales of units of AD drugs per quarter per 1 000 individuals aged 60 years and older.

© 2022 Ben Hassen C et al. *JAMA Health Forum.*

Piecewise regression was used to model the trends in sales over 10 years (2009 to 2019) using the R package “Segmented”.[5] Change in trends were tested using 1 to 7 knots to select the model with the lowest Akaike information criterion (AIC) along with fewest knots.

As data were aggregated by month and by age (France) or by quarter (Europe), no individual data were used for this study. Ethical approval was not required as only market data were used in this study.

© 2022 Ben Hassen C et al. *JAMA Health Forum.*

STROBE Statement—Checklist of items that should be included in reports of ***cross-sectional studies***

	Item No	Recommendation	Page No
**Title and abstract**	1	(*a*) Indicate the study's design with a commonly used term in the title or the abstract	2
		(*b*) Provide in the abstract an informative and balanced summary of what was done and what was found	
**Introduction**			
Background/rationale	2	Explain the scientific background and rationale for the investigation being reported	2
Objectives	3	State specific objectives, including any prespecified hypotheses	2
**Methods**			
Study design	4	Present key elements of study design early in the paper	2
Setting	5	Describe the setting, locations, and relevant dates, including periods of recruitment, exposure, follow-up, and data collection	2
Participants	6	(*a*) Give the eligibility criteria, and the sources and methods of selection of participants	2
Variables	7	Clearly define all outcomes, exposures, predictors, potential confounders, and effect modifiers. Give diagnostic criteria, if applicable	2
Data sources/ measurement	8*	For each variable of interest, give sources of data and details of methods of assessment (measurement). Describe comparability of assessment methods if there is more than one group	2
Bias	9	Describe any efforts to address potential sources of bias	Appendix, p1
Study size	10	Explain how the study size was arrived at	2
Quantitative variables	11	Explain how quantitative variables were handled in the analyses. If applicable, describe which groupings were chosen and why	2
Statistical methods	12	(*a*) Describe all statistical methods, including those used to control for confounding	2, Appendix p1
		(*b*) Describe any methods used to examine subgroups and interactions	NA
		(*c*) Explain how missing data were addressed	Appendix p1
		(*d*) If applicable, describe analytical methods taking account of sampling strategy	NA

© 2022 Ben Hassen C et al. *JAMA Health Forum.*

		(*e*) Describe any sensitivity analyses	NA
**Results**			
Participants	13*	(a) Report numbers of individuals at each stage of study—eg numbers potentially eligible, examined for eligibility, confirmed eligible, included in the study, completing follow-up, and analysed	NA
		(b) Give reasons for non-participation at each stage	NA
		(c) Consider use of a flow diagram	NA
Descriptive data	14*	(a) Give characteristics of study participants (eg demographic, clinical, social) and information on exposures and potential confounders	NA
		(b) Indicate number of participants with missing data for each variable of interest	NA
Outcome data	15*	Report numbers of outcome events or summary measures	3
Main results	16	(*a*) Give unadjusted estimates and, if applicable, confounder-adjusted estimates and their precision (eg, 95% confidence interval). Make clear which confounders were adjusted for and why they were included	3
		(*b*) Report category boundaries when continuous variables were categorized	3
		(*c*) If relevant, consider translating estimates of relative risk into absolute risk for a meaningful time period	NA
Other analyses	17	Report other analyses done—eg analyses of subgroups and interactions, and sensitivity analyses	NA
**Discussion**			
Key results	18	Summarise key results with reference to study objectives	3
Limitations	19	Discuss limitations of the study, taking into account sources of potential bias or imprecision. Discuss both direction and magnitude of any potential bias	4
Interpretation	20	Give a cautious overall interpretation of results considering objectives, limitations, multiplicity of analyses, results from similar studies, and other relevant evidence	3-4
Generalisability	21	Discuss the generalisability (external validity) of the study results	3-4
**Other information**			
Funding	22	Give the source of funding and the role of the funders for the present study and, if applicable, for the original study on which the present article is based	5

*Give information separately for exposed and unexposed groups.

© 2022 Ben Hassen C et al. *JAMA Health Forum.*

eReferences

1. Institut national de la statistique et des études économiques. https://www.insee.fr/fr/outil-interactif/5014911/pyramide.htm#! Accessed 01/03/2021.
2. Statistisches Bundesamt. https://service.destatis.de/bevoelkerungspyramide/. Accessed 05/07/2021.
3. Instituto Nacional de Estadística. https://www.ine.es/jaxiT3/Tabla.htm?t=34964&L=0. Accessed 05/07/2021.
4. Office of National Statistics. https://www.ons.gov.uk/peoplepopulationandcommunity/populationandmigration/populationestimates/articles/ukpopulationpyramidinteractive/2020-01-08. Accessed05/07/2021.
5. Muggeo V. Segmented: An R Package to Fit Regression Models With Broken-Line Relationships. *R News.* 2008;8:20-25.

© 2022 Ben Hassen C et al. *JAMA Health Forum.*
